# Consumer Perceptions and Acceptability of Traditional Dishes Prepared with Provitamin A-Biofortified Maize and Sweet Potato

**DOI:** 10.3390/nu11071577

**Published:** 2019-07-12

**Authors:** Laurencia Govender, Kirthee Pillay, Muthulisi Siwela, Albert Thembinkosi Modi, Tafadzwanashe Mabhaudhi

**Affiliations:** 1 Dietetics and Human Nutrition, School of Agricultural, Earth and Environmental Sciences, University of KwaZulu-Natal, Private Bag X01, Scottsville 3209, Pietermaritzburg 3201, South Africa; 2 Centre for Transformative Agricultural and Food Systems, School of Agricultural, Earth and Environmental Sciences, University of KwaZulu-Natal, Private Bag X01, Scottsville 3209, Pietermaritzburg 3201, South Africa

**Keywords:** bambara groundnut, crop biofortification, orange-fleshed sweet potato, provitamin A-biofortified maize, vitamin A deficiency

## Abstract

Vitamin A deficiency (VAD) is prevalent in South Africa, particularly among predominantly poor rural communities. Provitamin A (PVA)-biofortified crops could be used to address VAD; however, there are challenges of poor consumer acceptability. This study investigated the effect of replacing white maize and cream-fleshed sweet potato (CFSP) with PVA-biofortified maize and orange-fleshed sweet potato (OFSP), respectively, on consumer acceptability and perceptions of traditional dishes of rural communities in South Africa. Consumer acceptability of PVA-biofortified *phutu* (a crumbly maize porridge) served with either curried cabbage, chicken or bambara groundnut, separately, and boiled OFSP was evaluated by black South African adults (*n* = 120) using a five-point facial hedonic scale. Focus group discussions (FGDs) were conducted with 56 subjects recruited from the consumer panel to assess consumer perceptions of the food samples. The majority of the participants rated the composite dishes containing PVA-biofortified *phutu* as “4 = good” and the acceptability of the composite dishes varied significantly (*p* < 0.05). Compared to other age groups, the 50–59-year age group showed higher preference for white *phutu* and chicken curry, whereas the 30–39-year age group showed higher preference for PVA-biofortified *phutu* and chicken curry. The acceptability of OFSP and CFSP was similar. The study participants showed positive perceptions of the OFSP, as well as PVA-biofortified *phutu* if served with either curried chicken or cabbage. The findings suggest that PVA-biofortified maize and OFSP can replace white maize and CFSP, respectively, in selected traditional dishes of the rural communities studied to alleviate VAD.

## 1. Introduction

Worldwide, vitamin A deficiency (VAD) is a serious health problem affecting children and pregnant women [1], and is particularly prevalent among countries (including South Africa) in developing regions, especially sub-Saharan Africa (SSA) [2,3,4]. In South Africa, national studies showed that between 1994 and 2005, the VAD situation among children had worsened. The prevalence increased from 33.3 % in 1994 to 63.6% in 2005 [5,6]. Additionally, the National Food Consumption Survey-Fortification baseline (NFCS-FB-1) (2005) study reported that two in three women had VAD and six in ten women living in KwaZulu-Natal (KZN) had VAD. Overall, KZN had the second highest prevalence of VAD [5,6,7].

The more recent 2012 South African National Health and Nutrition Examination Survey (SANHANES-1) study found that VAD in children was still significantly high (43.6%) [8]. The 2012 SANHANES-1 study further reported that 45.5% of rural South Africans had VAD, and individuals with the lowest education levels were the most affected by VAD [8]. It is important to try to improve the vitamin A status of South Africans, especially children and vulnerable groups, as vitamin A is an essential micronutrient that has several physiological roles including immunity, vision and protein synthesis [9]. Vitamin A deficiency can result in an increased risk of mortality due to infections, thus emphasising the need to improve the vitamin A status of vulnerable groups [10].

One of the leading factors contributing to VAD is household food and nutrition insecurity [11]. In comparison to other regions in the world, Africa has the highest moderate (30%) and severe food insecurity (20%) [12]. Poor access to food, an element of food insecurity, is a common problem in South Africa, where 21.3% of households have severely inadequate or inadequate food access [13,14]. Although the general household survey (GHS) conducted in South Africa in 2017 showed a 1% decrease in the number of individuals with inadequate or severely inadequate access to food in comparison to the 2016 GHS [13,14], micronutrient malnutrition still remains a challenge. Suboptimal utilisation of available and accessible food is another dormant element of food insecurity in Africa. In South Africa, access to and utilisation of nutritious foods are a major problem, especially among impoverished individuals [13,15]. The consumption of nutritious foods promotes human development and enables an individual to perform the basic tasks needed for survival [16]. The South African government has implemented a number of strategies to reduce micronutrient deficiencies. These strategies include supplementation, food fortification, and dietary diversity. Despite the implementation of these strategies, there has been no significant improvement in the vitamin A status of the South African population [8,17,18,19]. Biofortification of staple crops is an emerging alternative complementary strategy to address VAD [20].

White maize and cream-fleshed sweet potato (CFSP) are two commonly grown and consumed staple crops in South Africa [21,22], and are therefore ideal for PVA-biofortification [21,22,23]. Three crops have been identified for provitamin A biofortification in Africa by HarvestPlus, namely cassava (*Manihot esculenta*), maize (*Zea mays*) and sweet potato (*Ipomoea batatas*) [24]. However, a number of studies conducted in Zambia, South Africa, Kenya and Ghana found that there were challenges with consumer acceptability of biofortified crops [25,26,27,28,29]. On the hand, other similar studies conducted in the same countries found that there were challenges with consumer acceptability of biofortified crops [30,31,32,33]. The poor acceptability of provitamin A-biofortified crops found in some studies may be attributed to the yellow/orange colour exhibited by carotenoids pigments found in the crops and the strong aroma and flavour of the biofortified crops, which are also attributed to the carotenoid pigments [34,35]. In addition, several studies have found that a stigma attached to the consumption of PVA-biofortified maize has negatively affected consumer acceptance of the biofortified maize [25,27,28,29]. There are a number of factors that have an impact on consumer acceptability of PVA-biofortified crops, such as gender, income, education, ethnicity, geographical location and background. Additionally, Pillay et al. found that the acceptability of PVA-biofortified maize varied with food type, which suggested that the acceptability of the biofortified maize could be improved by processing it into suitable food types [26].

Combining PVA-biofortified foods with other commonly consumed plant and animal food sources, could mask any undesirable sensory properties associated with biofortified crops and thereby increase their acceptability. For example, a study conducted by Amod et al. found that when PVA-biofortified maize porridge was combined with chicken stew its acceptability increased [36]. Additionally, PVA-biofortified composite dishes could improve the nutritional intake of vulnerable individuals [37]. A major problem in South Africa is poverty; it is a leading contributing factor to food and nutrition insecurity [38]. Currently (2019), a basic food basket in South Africa costs about $63, which is a 3% increase from last year [39]. This rise in food prices makes animal food sources unaffordable to VAD-vulnerable population groups, who are predominantly of low socioeconomic status. Thus, suitable food items should be sought from among the more affordable plant products for combining with PVA-biofortified foods to improve their acceptability. Plant products have also risen in price, but are generally cheaper than animal products. The most commonly consumed plant based foods in South Africa are maize (milled into maize meal) and green leafy vegetables—in the KZN province, maize (cooked maize meal) and legumes are leading [7,40]. Legumes could be suitable for combining with PVA-biofortified maize because they are rich in several nutrients [41], including protein. Starch-based cereal crops such as maize have limited amounts of lysine and tryptophan [42]. Lysine and tryptophan are essential amino acids as well. Lysine is required for the synthesis of peptide-hormones, antibodies, enzymes and muscle mass, whereas tryptophan is needed as it is a precursor for niacin, nicotinamide and serotonin [42]. Legumes generally contain higher amounts of lysine and tryptophan and have a lower content of sulfur-containing amino acids, especially methionine [42]. Thus, the consumption of a starch-based food such as maize together with a legume such as bambara groundnut would result in a balanced amino acid profile [43,44].

Bambara groundnut is an indigenous crop in Africa, it is well-adapted to and thrives in the agronomical marginal regions [45] where a significant proportion of vulnerable population groups live in SSA countries, including South Africa. Unlike other legumes, bambara groundnut contains the essential amino acid methionine [43,44,46]. Furthermore, bambara groundnut has been found to have high levels of essential fatty acids, vitamins and minerals [47]. Unfortunately, despite the fact that bambara groundnut is nutrient dense, it is generally underutilized as a food source in SSA due to several factors [48]. Bambara groundnut has hard-to-cook and hard-to-mill properties and exhibits antinutritional properties, a bitter taste and a strong beany flavour, which contribute to its limited utilization as a food source [49,50,51]. In South Africa, another limiting factor for the acceptability of bambara groundnut is the fact South Africans are generally not accustomed to it [52]. Although underutilized, a few studies have shown that bambara groundnut when prepared in some food types was positively accepted by consumers [52,53,54]. Further, heat processing methods such as roasting have been found to improve the taste and protein quality of bambara groundnut [54].

Unlike biofortified maize, for which mixed responses in consumer acceptability have been found, orange-fleshed sweet potato (OFSP) seems acceptable to consumers in South Africa, although data on its acceptability to different population groups are limited [30,33,55,56,57,58]. With regard to specific rural population groups in KZN province of South Africa, only one study has been conducted to assess consumer acceptance of PVA-biofortified maize foods served with other commonly consumed food items. Similarly, there are limited reports of consumer acceptability of OFSP in KZN. Thus, this study aimed to investigate the effect of replacing white maize and CFSP with PVA-biofortified maize and OFSP, respectively, on consumer acceptability and perceptions of traditional dishes of selected rural communities in KZN, South Africa.

## 2. Materials and Methods

### 2.1. Materials

A provitamin A-biofortified maize type (PVA A) and a white maize variety (control) (WE-3172) were selected for the study. The provitamin A-biofortified maize coded PVA A is an experimental type; it has not been commercialized, and hence does not have a variety name. The maize grain was produced by plant breeders from the University of KwaZulu-Natal (UKZN) and milled into maize for this study using methods described by Govender et al. [37]. Two varieties of sweet potato were selected for this study, OFSP (A45) and CFSP (A40). The bambara groundnut landrace and cabbage were purchased for the study.

### 2.2. Preparation of Food Products

All food dishes were prepared each morning during the sensory evaluation data collection in the Food Processing Laboratory in the Human Nutrition and Dietetics Department at UKZN, Pietermaritzburg. The food products were not made at the research sites, as there were no cooking facilities available. The cooked food samples were transported in insulating plastic containers closed with tight-fitting lids. Prior to this study, surveys were conducted to determine the commonly consumed food combinations and recipes were collected. Foods were prepared over two trial sessions a week prior to the pilot study to ensure that the recipes were accurate and culturally acceptable. These recipes were standardised for *phutu* (Supplementary Materials), cabbage curry (Supplementary Materials), chicken curry (Supplementary Materials) and sweet potato (Supplementary Materials). The bambara groundnut curry (Supplementary Materials) was cooked in the same manner as the beans curry (Supplementary Materials); however, extra water was added during the cooking process, and more time was needed to cook the bambara groundnut curry. This was due to the hard to cook properties of bambara groundnut [59]. The food dishes were tasted by black African males and females working at UKZN, who had a similar sociodemographic profile to the study participants to test for cultural acceptability. Although the UKZN workers were from similar areas as used in the main study, they would be at work during data collection, thus preventing them from participating in the main study. Table 1 gives a summary of the preparation methods of food items used in the current study.

### 2.3. Sensory Evaluation

A total of 120 black African adults comprising of males and females were randomly selected from Swayimane (29°25′50″ S–30°34′32″ E) and Umbumbulu (29°59′0″ S–30°42′0″ E) rural areas of KZN to participate in sensory evaluation of the study food samples. The sensory evaluation was conducted over two days (one day per site). Black African participants living in rural areas were selected for this study, as they are the population group at high risk for malnutrition [8,60]. A pilot study was carried out using 10 participants from the Swayimane area. The participants from the pilot study were excluded from the main study.

Participation was voluntary and panellists were allowed to leave the study at any point if they wished. Research assistants fluent in isiZulu were recruited from UKZN and trained prior to data collection. IsiZulu was the selected language as it is the local language spoken by the study participants. The participant number was given to maintain anonymity and for the research assistants to issue the correct sensory evaluation sheet to each participant.

Each of the food combinations was assigned a unique three-digit code obtained from a Table of Random Numbers [61]. The three-digit codes were known to the researcher, but not to the panellists, to prevent bias. The serving order of the food samples was determined by a Table of Random Permutations of Nine [61]. Each sample was carefully dished out so there was uniformity with portion size and appearance. Lawless and Heymann, reported that there are different variables to consider when deciding on a suitable food sample size. The researcher should consider the number of samples tested, what the mouthful of the specific product is, and what the study is trying to evaluate [62]. The quantities below were selected for the samples, as taste and texture were the only attributes that required tasting in order to rate it. The other attributes relied on other senses such as sight and smell. Each participant received ±75 mL of *phutu* with ±75 mL of cabbage curry, ±75 mL of *phutu* with ±75 mL chicken curry and ±75 mL of *phutu* with ±75 mL of bambara groundnut curry. Participants were also given a quarter piece (±25 g) of sweet potato in a 250 mL polystyrene cup, separately.

Additionally, the paired preference test also required participants to taste and compare the samples. The food sample quantities were tested at the pilot study to determine if the participants required more of either food item to evaluate the dishes and thereafter the quantity was selected for the main study. At the pilot study, the participants received ±30 mL of *phutu* and each of the curries, which was not enough for testing, thus the quantity was increased for the main study. The samples were warmed for ten seconds prior to being served and the temperature checked to ensure that it was warm before being served to each participant. This was done to ensure that the samples were not cold as cold samples could negatively affect the outcome of the sensory evaluation.

Each of the panellists was escorted to their station by a research assistant. Panellists were told not to communicate with one another during the sensory evaluation. A separating board divided the panellists, so that they could not communicate with each other during the sensory evaluation session. Each panellist was provided with a pen, a cup of water to rinse the palate between samples and sensory evaluation questionnaires (five-point hedonic facial scale and paired preference test) developed in isiZulu, the local language in KZN. The English versions of the sensory evaluation questionnaires (five-point hedonic facial scale and paired preference test were translated into isiZulu, and then back to English by two separate translators proficient in both languages, to ensure that the translation was accurate). The five-point hedonic facial scale (1 = very bad; 5 = very good) was used so that the semi-literate/illiterate participants could record their responses. The five-point hedonic facial scale tested the sensory attributes (taste, texture, aroma, colour and overall acceptability) of six maize combination food samples (white *phutu* and cabbage curry; PVA-biofortified *phutu* and cabbage curry; white *phutu* and chicken curry; PVA-biofortified *phutu* and chicken curry; white *phutu* and bambara groundnut curry; PVA-biofortified *phutu* and bambara groundnut curry). The same scale was used to test the sensory attributes of two sweet potato samples (boiled CFSP and boiled OFSP). The maximum number of samples that should be tasted at a time is six for a full description analysis and a maximum of ten samples if testing less than 10 sensory attributes [63]. For the purpose of the study, the six maize dishes were tasted first and thereafter the two sweet potato dishes. One dish was given to the participant at a time. Participants were required to put a cross over the face that they felt best described the attributes of the sample that was tasted. The research assistant explained each sensory attribute to the participant before the samples were evaluated, so that the participant knew what was meant by each of the five attributes.

Each panellist completed the paired preference test after the panellists tasted all eight-food samples. Samples of white *phutu* combinations were compared with yellow *phutu* combinations and CFSP was compared with OFSP. The paired preference test is used to investigate the preference after tasting two products and adapted versions are simple to use for semi-literate/illiterate participants [62]. The paired preference test in this study used the three-digit code that was allocated to the two samples during the initial sensory evaluation using the five-point facial hedonic scale. The participant was requested to put a circle around the three-digit number that appeared on the cup the sample that they preferred. The paired preference test conducted alone does not give a true reflection on whether or not a sample is liked, as both samples may be disliked, and one may be more preferred [62]. However, this test helps to validate the results from the sensory evaluation using a five-point hedonic facial scale, which was done in this study.

### 2.4. Focus Group Discussions

Fifty-six black African adults comprising males and females from Swayimane and Umbumbulu were randomly selected from the sensory evaluation panel to participate in the FDGs. On completion of the sensory evaluation survey, 30 participants were randomly invited from each research site to participate in the FGDs. Only 56 participants from both areas agreed to participate in the FGDs. The participants were divided into five groups of between 7 and 10 participants each. This was decided as the ideal size for a focus group discussion is between 6 and 10 participants [48,64,65]. A pilot study was conducted using the sensory evaluation participants in the Swayimane area prior to the main study to test the FGDs questions. The FGDs were facilitated by two trained research assistants (one male and one female), who were fluent in isiZulu and had experience with conducting focus group discussions. The research assistants alternated facilitating the FGDs. The FGDs questions were developed in advance from the themes that were identified. The FGD questions were first formulated in English by the researcher and then translated into isiZulu by two isiZulu-speaking individuals. The FGD questions were checked by an individual who has experience working with focus groups and the questions were tested on a group of black African males and females working at the UKZN, who had a similar sociodemographic profile to the study participants to test for appropriateness. Two research assistants were involved in the translation to ensure that the questions were translated accurately into isiZulu and the intended questions were not lost in translation. The FGD questions were tested in a pilot study and no changes were made to the questions. A digital voice recorder was used to record the FDGs as all participants had consented to the use of the voice recorder. The recordings were later translated into English by both focus group discussion facilitators. The translated recordings were cross-checked by an isiZulu-speaking person against the English translation, for accuracy.

### 2.5. Ethics Approval

Ethical approval was obtained from the UKZN, Humanities and Social Science Ethics Committee (HSS/0256/016D). Gatekeeper’s permission was obtained to conduct the study at the research sites. Each panellist provided written consent prior to participating in the sensory evaluation. The consent form was read to the participants in isiZulu so that all participants understood the contents of the consent form. All participants were able to sign the consent form. The participants were shown where to sign or initial on the consent form if they understood what was explained. If they did not understand something, it was re-explained and they signed once they understood. The consent form also allowed participants to grant permission to be photographed and audio and video recorded.

### 2.6. Statistical Analysis

Data from the sensory evaluation questionnaires were analysed using the Statistical Package for Social Sciences (SPSS version 25.0 SPSS Inc, Chicago, IL, USA) at the 5% level of significance. The Friedman’s test—a nonparametric statistical test—was used to test for significant differences in sensory attributes across the *phutu* combinations. The specific differences were then analysed using the Wilcoxon test for the *phutu* combinations and two varieties of sweet potato. The independent samples *t*-test was used to determine significant differences across gender for the average sensory attributes of the food samples. Analysis of variance (ANOVA) was used to determine significant differences between different age groups of the sensory attributes for all food samples. The Welch test was used when conditions of the ANOVA test were not met. The paired preference results was analysed using a Pearson chi-square test. The responses from the FGDs were subjected to thematic content analysis. Verbatim comments from the FGDs were extracted from the voice recorder and translated from isiZulu into English. Data from the FGDs and the notes were coded. Similar coded ideas were then arranged into appropriate themes. Thereafter, a discussion was written for each theme.

## 3. Results

### 3.1. Sample Characteristics

The majority of participants that participated in the sensory evaluation and FGDs were female. (Table 2 and Table 3). This was expected as in rural areas; males usually work away from home and females stay at home and care for the family. Although, most of the sensory evaluation participants were above 60 years of age (*n* = 29), the majority of the FGDs comprised of participants aged 40-49 years (*n* = 16).

### 3.2. Sensory Evaluation

The panellists did not experience any adverse effects after tasting the PVA-biofortified composite dishes and boiled OFSP. The panellists rated the sensory attributes (taste, texture, aroma, colour and overall acceptability) of all eight dishes as ‘good’. Table 4 and Table 5 indicate the percentages of panellists who gave the different ratings for the sensory attributes and composite dishes evaluated. Most of the study participants rated the taste, texture and aroma of PVA *phutu* and chicken curry as “good” compared with white *phutu* and chicken curry (control). The texture and colour of PVA *phutu* and cabbage curry was rated “good” by more study participants in comparison to white *phutu* and cabbage curry (control). The PVA *phutu* and bambara groundnut combination was rated as “good” for the overall acceptability when compared with white *phutu* and bambara groundnut curry (control). When comparing the composite dishes made with PVA *phutu*, the PVA *phutu* and chicken curry combination was rated “good” by most study participants for all five sensory attributes. The PVA *phutu* and cabbage curry combination was rated as “good” by most participants for taste, texture, aroma and colour in comparison to PVA *phutu* and bambara groundnut curry, however the overall acceptability of PVA *phutu* and bambara groundnut curry was rated “good” by more participants. Orange-fleshed sweet potato was rated “good” by most participants for taste and texture in comparison to CFSP, however the same number of participants rated OFSP and CFSP as “good”.

The mean scores for the sensory evaluation of the composite dishes and sweet potato are presented in Table 6. Results from the Friedman test showed that there was a significant difference in taste, texture, aroma, colour and overall acceptability (*p* < 0.05) across the dishes that included *phutu*. In order to determine the specific significant differences between the composite dishes for each of the sensory attributes, the Wilcoxon test was applied. Results are summarised in Table 7 and Table 8. The sensory attributes taste, texture aroma, colour and overall acceptability of PVA *phutu* combined with chicken curry were rated similarly (*p* > 0.05) by participants in comparison to white *phutu* served with chicken curry. Provitamin A-biofortified *phutu* and cabbage curry combination had a similar rating (*p* > 0.05) for all five sensory attributes in comparison to white *phutu* and cabbage curry. The PVA *phutu* and bambara groundnut curry had a significantly lower rating (*p* < 0.05) for taste in comparison to white *phutu* and bambara groundnut curry (control); however, both composite dishes were rated similarly (*p* > 0.05) for the other sensory attributes. When comparing the PVA composite dishes, it was found that when PVA *phutu* was combined with either curried cabbage or chicken, the taste, texture and overall acceptability was rated better (*p* < 0.05) than that of PVA *phutu* and bambara groundnut curry. Although the PVA *phutu* and chicken curry had a significantly (*p* < 0.05) better rating for colour in comparison to PVA *phutu* and cabbage curry, the PVA *phutu* and cabbage combination was rated higher for colour (*p* < 0.05) in comparison to PVA *phutu* and bambara groundnut curry. The Wilcoxon test found that the two varieties of sweet potato were rated similarly (*p* > 0.05) for taste, texture, aroma, colour and overall acceptability (Table 6).

Results from the independent samples *t*-test found that there were no significant differences (*p* > 0.05) across gender for the average sensory attributes of all eight dishes. From the *phutu* composite dishes, the male participants rated the taste of white *phutu* and cabbage curry the highest, whereas females rated the PVA *phutu* and chicken curry the highest. Both males and females least liked the taste of the PVA *phutu* and bambara groundnut combination. The male participates liked the texture of the composite dishes that contained chicken curry, whereas female participants preferred the white *phutu* and chicken curry combination. Female participants preferred the aroma and colour of PVA *phutu* and chicken curry and the male participants preferred the aroma colour of white *phutu* and chicken curry. The overall acceptability of white *phutu* and chicken curry and PVA *phutu* and chicken curry was rated the same by males, however, female participants preferred the PVA *phutu* and chicken curry combination. The taste and texture of CFSP was liked by both males and females in comparison to OFSP. Female participants preferred the aroma of CFSP to OFSP, while the male participants rated the aroma of both varieties of sweet potato the same. Conversely, females preferred the colour of OFSP to CFSP, while the male participants rated the colour of OFSP and CFSP the same. The overall acceptability of OFSP was rated higher by males and lower by females in comparison to CFSP.

Results from applying ANOVA indicated a significant difference (*p* > 0.05) in the average sensory attribute taste for white *phutu* and cabbage curry and white *phutu* and bambara groundnut curry across certain age categories (Table 9). The Welch test indicated that there was a significant difference (*p* < 0.05) between certain age categories and the sensory properties aroma and overall acceptability for white *phutu* and bambara groundnut curry (Table 9). There was no significant difference (*p* > 0.05) in the acceptability ratings of PVA-biofortified composite dishes across age groups. The taste of the PVA *phutu* and chicken curry, PVA *phutu* and cabbage curry were preferred by participants aged 30–39 years and 40–49 years, respectively. The 40–49 age group preferred the texture and aroma of the PVA *phutu* and chicken curry and PVA *phutu* and cabbage curry in comparison to the other age groups. The colour was preferred by both the 30–39 and 50–59 years age group. The participants older than 60 years preferred the colour and overall acceptability of the PVA *phutu* and cabbage combination in comparison to the other age groups. The taste, texture, aroma, colour and overall acceptability of PVA *phutu* and bambara groundnut curry was rated higher by the above 60 year olds in comparison to other age groups. The 20–29 year old participants preferred the taste, texture, aroma, colour and overall acceptability of OFSP in comparison to the other age groups.

The paired preference results are presented in Table 10 and Table 11. There was no statistical significance noted for the preference of PVA-biofortified and non-PVA-biofortified food combinations between males and females (*p* > 0.05). Although not statistically significant, males from this sample preferred the white *phutu* and chicken curry (*n* =19; 56%), white *phutu* and bambara groundnut curry (*n* = 19; 56%) and OFSP (*n* = 20; 59%). The females participants preferred the provitamin A-biofortified *phutu* and chicken curry (*n* = 46; 54%), white *phutu* and cabbage curry (*n* = 44; 51%), white *phutu* and bambara groundnut curry (*n* = 47; 55%) and OFSP (*n* = 65; 54%).

The participants aged 30–39 years who preferred provitamin A-biofortified *phutu* and chicken (*n* = 17; 71%) was significantly (*p* < 0.05) higher than the other age groups. Moreover, participants aged 50–59 years who preferred white *phutu* and chicken (*n* = 17; 68%), was statistically significantly higher than participants from the other age groups. There was a tendency for all age groups to prefer white *phutu* with cabbage, white *phutu* with bambara groundnut and OFSP, although this was not statistically significant (*p* > 0.05).

### 3.3. Focus Group Discussions

There were both positive and negative responses to the PVA-biofortified *phutu* and bambara groundnut combination; however, overall the participants had positive perceptions about the sensory properties of the PVA-biofortified food combinations and OFSP. The participants offered suggestions as to how the meals could be prepared to increase the acceptability. The participants were not asked how to improve the meals however, participants offered suggestions during the discussion. The participants expressed a willingness to purchase PVA-biofortified maize and sweet potato if it was available. The participants were also keen to grow their own biofortified produce if seeds were accessible. The results are presented in Table 12.

## 4. Discussion

Malnutrition is the leading contributor to the global disease burden [66,67,68]. South Africa is faced with the double burden of malnutrition. There are many interventions that focus on under and over nutrition in vulnerable population groups; however, micronutrient deficiencies are still prevalent. Although the fortification of maize meal and wheat flour was legislated in SA in October 2003, the accessibility of these commercially fortified foods to rural households remains questionable [19]. Provitamin A-biofortified crops improve the nutrient content of foods, but their acceptability to target consumers should be improved through research. Thus, this study aimed to investigate the effect of replacing white maize and CFSP with PVA-biofortified maize and OFSP, respectively, on consumer acceptability and perceptions of traditional dishes of selected rural communities in KZN, South Africa.

The study results are encouraging as the sensory attributes taste, texture, aroma, colour and overall acceptability were rated as good by most study participants for all of the PVA *phutu* composite dishes. Over millennia ago, in rural South African communities, indigenous and traditional crops were the main source of foods. However, with urbanisation there has been a shift from traditional foods to more western foods and these indigenous and traditional foods are less consumed [69]. The older generation have some knowledge of bambara groundnut [52], which was confirmed in the FGDs. The older generation (above 60 years) rated the overall acceptability of *phutu* and bambara groundnut curry better than the younger generation (20–29 years). Chowdhury et al. reported that introducing an unfamiliar product could negatively affect consumer acceptance [30], which could be the case in this study among the younger generation for the *phutu* and bambara groundnut curry combination. Although bambara groundnut was an unfamiliar food crop, it was introduced and investigated in this study as it could be a cheaper alternative to animal food sources. To overcome this study limitation, crops like bambara groundnut could be included in the meals used in school feeding programs. This will not only improve the nutritional intake of these children but would result in earlier exposure to this crop. Early and frequent exposure to a food item improves the acceptance of the food item [35]. Another suggestion would be to prepare bambara groundnut into different food types using preparation methods that have shown to improve acceptance, such as roasting. A study conducted by Oyeyinka et al. found that bambara groundnut made into a pureed infant complementary food was acceptable to caregivers [52]. Another study conducted in Nigeria using a snack made from bambara groundnut flour found that the aroma, colour, crunchiness and overall acceptability were higher than those of the control made with cowpea [53]. Additionally, Okafor et al. found that substituting roasted bambara groundnut at different substitution levels for wheat flour in biscuits, had a high sensory rating for the attributes investigated. However, the flavour of the biscuits was similar to that of the control for up to 70% substitution [54]. These studies further confirm that the way in which a food item is prepared and the geographic location of consumers influence its acceptability.

As alluded to earlier, poverty is a problem and impoverished individuals rely on, in some instances, social grants which are used as the sole source of income for purchasing food [70]. Hence, there is a lack of dietary diversity and a high reliance on starch-based foods, such as maize meal as they are cheaper [71,72]. Thus, it is important to introduce impoverished individuals to affordable nutritious food alternatives such as bambara groundnut that can be consumed together with cooked maize meal. It is important to note that an individual’s background, traditions, socioeconomic standing and geographical location are important factors that influence the types of foods consumed and the preparation methods used.

The participants from the present study were unfamiliar with bambara groundnut and the younger generation lacked basic knowledge about this crop. This emphasises the need for education on the nutritional benefits of bambara groundnut and methods for preparation, in order to improve exposure and acceptance of this underutilised crop. It is noteworthy that this study did not educate participants about the composite dishes prior to the sensory evaluation and FGDs. Future studies could investigate the impact of nutritional education on consumer acceptability.

The acceptance of PVA-biofortified maize has been previously investigated by several authors [26,29,33,73,74,75]. Provitamin A-biofortified maize has been found to have an undesirable colour. The grain colour changes from white to either yellow or orange due to the carotenoid pigments [73,76], thus contributing to poor acceptability [28]. However, the change did not hinder colour acceptability of the yellow *phutu* and OFSP in this study. A number of studies have investigated the preference of PVA-biofortified maize to white maize and found mixed responses [25,73,74,75,76]. However, there is paucity of information regarding the preference of PVA-biofortified *phutu* composite dishes compared to corresponding white maize composite dishes. The PVA *phutu* and chicken curry combinations were well accepted in the current study. This result was similar to the results obtained by Amod et al. who investigated the sensory acceptability of yellow *phutu* consumed together with chicken stew [36]. The authors found that the combination of yellow *phutu* and chicken was well accepted by caregivers attending the paediatric outpatient department at Edendale Hospital in KZN [36]. The participants of the study conducted by Amod et al. were similar to the current study participants, as Edendale hospital mainly services individuals living in surrounding rural areas. In the current study, it was reported that the aroma and colour of the chicken and yellow *phutu* were well-accepted. These findings suggest that the combination of chicken curry and yellow *phutu* was well-accepted by study participants and could help improve the vitamin A intake in vulnerable groups. However, as mentioned earlier, animal food sources are not affordable. Thus, bambara groundnut should be considered not only as a nutritious alternative but also a viable production option in these areas.

A number of studies conducted on OFSP have shown positive responses from participants, despite the orange colour [30,55,58,77,78]. A study conducted by Pillay et al. on infant caregivers found that a complementary food made with OFSP was well accepted by the caregivers [55]. Additionally, another study that investigated the acceptance of OFSP by caregivers, reported that the OFSP was preferred to pale-fleshed sweet potato for all sensory attributes investigated [58]. Moreover, a study conducted in Uganda found that the deep orange coloured sweet potato was preferred over yellow or white sweet potato [30]. Although not statistically significant, numerically, OFSP was preferred to CFSP. This could be due to the sweet taste and colour of OFSP. The results from this study are encouraging as they suggest that there is a potential to use OFSP in some rural areas of KZN similar to the study sites, to improve the vitamin A status of vulnerable individuals.

Consumption of biofortified crops such as maize and OFSP and the consumption of an indigenous crop like bambara groundnut could potentially increase the dietary diversity of impoverished individuals and improve nutritional status [79]. However, bambara groundnut should be introduced in a different cooked form rather than a curry to improve acceptability. For this study, bambara groundnut was cooked in a similar manner to bean curry to investigate the acceptance. The younger generation were unaccustomed to bambara groundnut and the older generation that were familiar with it, enjoyed the taste. Acceptance of bambara groundnut could be improved if it is cooked together with *phutu* or mealie meal or milled into a flour and used to prepare other products. There is a need to further investigate the sensory acceptability of combining other cooked PVA-biofortified maize foods such as stiff pap, mealie meal porridge, *amaheu, isigwaqane or isijingi,* with commonly consumed food items in rural KZN, and other provinces within South Africa. Furthermore, future studies could explore the impact of education on the nutritional benefits of these crops and the acceptance, perception and consumption of these crops.

The FGDs results correlate with the results obtained from the sensory evaluation as the FDGs participants had positive perceptions of the combination of PVA-biofortified *phutu* with chicken and cabbage; however, there were mixed responses with regard to PVA-biofortified *phutu*, and bambara groundnut curry. This result was not surprising, as bambara groundnut is an indigenous nutritious crop; however, it is not normally consumed in rural areas of KZN [80]. The older generation of participants perceived the *phutu* and bambara groundnut combination as something the younger generation would not like, as they are not accustomed to it. A study conducted by Oyeyinka et al. found that the older generation was familiar with the preparation of bambara groundnut [52]. This study further identified that a lack of knowledge may be a reason for the underutilisation of this crop [52]. It is important to provide knowledge, especially to the younger generation on the nutritional benefits of consuming this crop as well as good agricultural practices to produce this crop. Knowing the nutritional value of a particular food item could improve the acceptance of that specific item [31]. Furthermore, indigenous crops such as bambara groundnut should be promoted to local farmers to improve the production and access to these crops. These crops could become cash crops and further provide not only nutrients but income for the local farmers.

Participants from the FGDs perceived the foods to be culturally acceptable and familiar; however, they made suggestions as to what should be changed to improve the combinations. A few male participants from the FGDs suggested that PVA-biofortified maize should be cooked into stiff pap and served with chicken curry instead of *phutu*. A survey, which was conducted at the start of this study, found that 84.4% of the study participants consumed *phutu*, whereas only 42.4% consumed stiff pap. From the combinations investigated, more participants preferred *phutu* and chicken curry (n = 27), rather than stiff pap and chicken curry (n = 20). Although, it was suggested that the PVA-biofortified maize should be cooked into stiff pap and served with chicken curry, the *phutu* combination was still well perceived. Future studies should investigate consumer perceptions of cooking PVA-biofortified maize into food forms other than *phutu*, and served with commonly consumed foods. This would offer a variety of more acceptable foods that individuals could consume and as a result possibly improve the nutritional status of vulnerable individuals, particularly vitamin A.

The OFSP prepared for the sensory evaluation in this study seemed to retain water. Some of the participants mentioned that they would have enjoyed it more if it were boiled for longer in less water. The same amount of water and time taken for straining was used to cook the CFSP. A possible reason for excess water content could be attributed to a relatively lower dry matter content of the OFSP. The genotypes found in the different varieties of OFSP influence the dry matter content and affect consumer acceptability [81]. Some participants suggested that the bambara groundnut curry needed to be cooked for a longer period of time and that less water should be used when cooking OFSP. Further studies could investigate the acceptance of OFSP when prepared with different amounts of water and cooking times to improve acceptability. If OFSP is well accepted and consumed in areas where VAD is a significant problem, it has the potential to improve the vitamin A status of these individuals.

Many study participants perceived the OFSP to be butternut due to the orange colour, sweet taste and visual appeal, which was similar to the findings of a study conducted by Pillay et al. on the acceptance of OFSP [55]. Moreover, other studies have also reported that OFSP has been compared to pumpkin [58,78]. Although OFSP may be unfamiliar to some individuals, it resembles other familiar food items. Participants can therefore relate to it and are more likely to consume it. Generally, individuals are more inclined to consume foods that are familiar to them [30]. The participants mentioned that they would not have enjoyed yellow *phutu* on its own. This was possibly due to the undesirable sensory properties of biofortified foods and some participants being unfamiliar with it [34,35]. This further suggests that if yellow maize is consumed with another food item it may mask the undesirable sensory changes noted with biofortified foods, thus increasing its acceptance. Participants’ also indicated that the yellow *phutu* was appealing when served with chicken curry or bambara groundnut curry. These results were consistent with the study conducted by Amod et al. [36].

Many participants expressed that yellow maize was used as an animal feed or during drought. This was similarly expressed in other studies [25,36,74,76]. However, study participants expressed a willingness to grow and purchase yellow maize and OFSP if seeds were made available or they could be found in shops. Although PVA-biofortified foods have been found to be less acceptable by some studies, foods investigated in the study were positively perceived by most of the FGD participants. Therefore, PVA-biofortified maize and OFSP could replace white maize and CFSP, respectively, as these foods are rich in vitamin A and thus could contribute to addressing VAD, which is prevalent in rural areas of South Africa. However, there is a need to provide education on the health benefits of these crops especially to the younger generation who are not accustomed to these crops, to improve acceptance.

## 5. Conclusions

PVA-biofortified foods served on their own have been well-accepted in some studies, while other studies have found a poor acceptance due to several factors. The results of this study were encouraging as foods investigated in this study were positively perceived by the majority of the study participants. This study indicates that the undesirable properties of PVA-biofortified foods that were found in other studies can be masked by serving it with another commonly consumed food item, thus improving the acceptance. Even though *phutu* and bambara groundnut curry were not as preferred in comparison to the other combined meals investigated, it was rated as ‘good’ for all the sensory attributes. This indicated that although it was least preferred in comparison to the other combinations, if served on its own it would be acceptable to consumers. *Phutu* and chicken curry was the most preferred combined dish, however, it contains animal protein which is not always affordable to impoverished individuals. Bambara groundnut can be used as an alternative affordable plant-based protein source; however, the acceptance needs to be further investigated in other food products such as incorporating the bambara groundnut and *phutu* during cooking, addition of bambara groundnut to maize meal to make a traditional drink or serving it with another form of cooked maize meal. Overall, it appears that PVA-biofortified maize combined with chicken curry, cabbage curry, bambara groundnut curry and boiled OFSP have the potential to be used as healthy alternatives in rural KZN. However, more studies need to be conducted on trying to improve the exposure to and acceptance of provitamin A-biofortified maize and bambara groundnut together, especially to the younger generation. The bambara groundnut and PVA *phutu* combination could be used in school feeding programs as a cheaper alternative to animal food sources. Not only will it improve the nutritional intake of young children, but provide exposure to these crops to consumers at a young age. Additionally, more education needs to be conducted on the nutritional benefits of PVA-biofortified crops and bambara groundnut, especially for the younger generation as many of the younger generation are not familiar with these crops. Moreover, there should be the promotion of PVA-biofortified crops (maize and sweet potato) and bambara groundnut to local farmers. Local farmers should be educated on the production of these crops and possibly given or sold seeds at a reduced cost. This will result in an increased production of these crops by farmers, which could lead to improved consumption. This study suggests that PVA-biofortified maize and OFSP could be incorporated into the diets of the rural communities studied to contribute to combating VAD, which is a major problem in South Africa and sub-Saharan Africa.

## Figures and Tables

**Table 1:** Methods of food items used in the study.

Food Item	Description
White maize *phutu* (control)	A popular crumbly maize porridge and traditional maize food product in KZN.
PVA maize *phutu* (test sample)	Crumbly maize porridge made with PVA maize in place of white maize.
CFSP (control)	The traditional, popular CFSP was boiled as is commonly done by the studied communities.
OFSP (test sample)	The traditional, popular CFSP was replaced by the OFSP, which was boiled in the same manner as the CFSP.
Cabbage curry	Cooked following a traditional recipe obtained from study participants, with oil Raja spice, onions, shredded cabbage, water, salt and a stock cube.
Chicken curry	Cooked following a traditional recipe obtained from study participants with oil, Raja spice, onions, cut chicken pieces, water, salt and two stock cubes.
Bambara groundnut curry	Cooked following a modified traditional recipe obtained from study participants for dry beans curry, which was prepared with oil, Raja spice, onions, bambara groundnut (soaked overnight), water, salt, bicarbonate of soda and a stock cube.

PVA = pro-Vitamin A; CFSP = cream-fleshed sweet potato; OFSP = orange-fleshed sweet potato.

**Table 2:** Characteristics of sensory participants.

Characteristic	n (%) *
**Gender**	
Males	34 (28.3)
Females	86 (71.7)
**Age group (years)**	
20–29	14 (11.7)
30–39	24 (20.0)
40–49	28 (23.3)
50–59	25 (20.8)
60+	29 (24.2)

* Percentage of sample calculated using total sample (*n* = 120).

**Table 3:** Characteristics of the focus group discussion (FGD) participants.

Characteristic	*n* (%) *
**Gender**	
Males	16 (28.6)
Females	40 (71.4)
**Age group (years)**	
20–29	4 (7.1)
30–39	12 (21.4)
40–49	16 (28.6)
50–59	15 (26.8)
60+	9 (16.1)

* Percentage of sample calculated using total sample (*n* = 56).

**Table 4:** Number and percentages of panellists who gave the different ratings for the sensory attributes evaluated (*n* = 120).

Composite Dishes	Rating	Taste	Texture	Aroma	Colour	Overall Acceptability
White *phutu* and chicken curry	Very bad	1 ^a^ (0.8) ^b^	2 (1.7)	0 (0.0)	0 (0.0)	0 (0.0)
	Bad	11 (9.2)	12 (10.0)	4 (3.3)	4 (3.3)	1 (0.8)
	Average	15 (12.5)	13 (10.8)	14 (11.7)	20 (16.7)	12 (10.0)
	Good	65 (54.2)	65 (54.2)	74 (61.7)	78 (65.0)	83 (69.2)
	Very good	28 (23.3)	28 (23.3)	28 (23.3)	18 (15.0)	24 (20.0)
PVA *phutu* and chicken curry	Very bad	1 (0.8)	2 (1.7)	1 (0.8)	0 (0.0)	0 (0.0)
	Bad	8 (6.7)	13 (10.8)	4 (3.3)	10 (8.3)	6 (5.0)
	Average	11 (9.2)	8 (6.7)	12 (10.0)	10 (8.3)	6 (5.0)
	Good	72 (60.0)	75 (62.5)	81 (67.5)	67 (55.8)	78 (65.0)
	Very good	28 (23.3)	22 (18.3)	22 (18.3)	33 (27.5)	30 (25.0)
White *phutu* and cabbage curry	Very bad	2 (1.7)	2 (1.7)	0 (0.0)	0 (0.0)	0 (0.0)
	Bad	11 (9.2)	13 (10.8)	10 (8.3)	9 (7.5)	7 (5.8)
	Average	15 (12.5)	29 (24.2)	22 (18.3)	30 (25.0)	15 (12.5)
	Good	65 (54.2)	53 (44.2)	66 (55.0)	62 (51.7)	77 (64.2)
	Very good	27 (22.5)	23 (19.2)	22 (18.3)	19 (15.8)	21 (17.5)
PVA *phutu* and cabbage curry	Very bad	0 (0.0)	1 (0.8)	0 (0.0)	0 (0.0)	0 (0.0)
	Bad	9 (7.5)	12 (10.0)	8 (6.7)	9 (7.5)	8 (6.7)
	Average	24 (20.0)	26 (21.7)	24 (20.0)	16 (13.3)	14 (11.7)
	Good	62 (51.7)	57 (47.5)	64 (53.3)	69 (57.5)	60 (50.0)
	Very good	25 (20.8)	24 (20.0)	24 (20.0)	26 (21.7)	38 (31.7)
White *phutu* and bambara groundnut curry	Very bad	12 (10.0)	10 (8.3)	2 (1.7)	1 (0.8)	6 (5.0)
	Bad	18 (15.0)	22 (18.3)	18 (15.0)	12 (10.0)	13 (10.8)
	Average	10 (8.3)	13 (10.8)	22 (18.3)	23 (19.2)	11 (9.2)
	Good	62 (51.7)	62 (51.7)	68 (56.7)	71 (59.2)	70 (58.3)
	Very good	18 (15.0)	13 (10.8)	10 (8.3)	13 (10.8)	20 (16.7)
PVA *phutu* and bambara groundnut curry	Very bad	13 (10.8)	11 (9.2)	6 (5.0)	3 (2.5)	4 (3.3)
	Bad	20 (16.7)	20 (16.7)	14 (11.7)	16 (13.3)	17 (14.2)
	Average	20 (16.7)	16 (13.3)	26 (21.7)	22 (18.3)	11 (9.2)
	Good	60 (50.0)	55 (45.8)	62 (51.7)	65 (54.2)	73 (60.8)
	Very good	7 (5.8)	18 (15.0)	12 (10.0)	14 (11.7)	15 (12.5)

^a^ Number of subjects; ^b^ Percentage of total number of participants; PVA = Provitamin A; Acceptability rating 1–5: 1 = very bad; 5 = very good.

**Table 5:** Number and percentages of panellists who gave the different ratings for the sensory attributes evaluated (*n* = 120).

Sweet Potato	Rating	Taste	Texture	Aroma	Colour	Overall Acceptability
CFSP	Very bad	1^a^ (0.8) ^b^	0 (0.0)	0 (0.0)	2 (1.7)	2 (1.7)
	Bad	9 (7.5)	8 (6.7)	6 (5.0)	6 (5.0)	2 (1.7)
	Average	3 (2.5)	16 (13.3)	24 (20.0)	20 (16.7)	10 (8.3)
	Good	61 (50.8)	50 (41.7)	63 (52.5)	67 (55.8)	70 (58.3)
	Very good	46 (38.3)	46 (38.3)	27 (22.5)	25 (20.8)	36 (30.0)
OFSP	Very bad	2 (1.7)	2 (1.7)	1 (0.8)	4 (3.3)	3 (2.5)
	Bad	7 (5.8)	11 (9.2)	12 (10.0)	7 (5.8)	4 (3.3)
	Average	6 (5.0)	8 (6.7)	26 (21.7)	13 (10.8)	9 (7.5)
	Good	62 (51.7)	60 (50.0)	58 (48.3)	67 (55.8)	62 (51.7)
	Very good	43 (35.8)	39 (32.5)	23 (19.2)	29 (24.2)	42 (35.0)

^a^ Number of subjects; ^b^ Percentage of total number of participants; CFSP = Cream-fleshed sweet potato; OFSP = Orange-fleshed sweet potato; Acceptability rating 1–5: 1 = very bad; 5 = very good.

**Table 6:** Mean scores for the sensory evaluation of PVA-biofortified maize and OFSP dishes compared with the control white maize and CFSP dishes (*n* = 120)**.**

Sensory Attributes	Taste	Texture	Aroma	Colour	Overall Acceptability
**Composite dishes**					
White *phutu* and chicken curry	3.9 ^a^ (0.9) ^b^	3.9 (0.9)	4.1 (0.7)	3.9 (0.7)	4.1 (0.6)
PVA *phutu* and chicken curry	4.0 (0.8)	3.9 (0.9)	4.0 (0.7)	4.0 (0.8)	4.1 (0.7)
White *phutu* and cabbage curry	3.9 (0.9)	3.7 (1.0)	3.8 (0.8)	3.8 (0.8)	3.9 (0.7)
PVA *phutu* and cabbage curry	3.9 (0.8)	3.8 (0.9)	3.9 (0.8)	3.9 (0.8)	4.1 (0.8)
White *phutu* and bambara groundnut curry	3.5 (1.2)	3.4 (1.2)	3.6 (0.9)	3.7 (0.8)	3.7 (1.1)
PVA *phutu* and bambara groundnut curry	3.2 (1.1)	3.4 (1.2)	3.5 (1.0)	3.6 (0.9)	3.7 (1.0)
*p*-value ^c^	<0.05	<0.05	<0.05	<0.05	<0.05
**Sweet potato**					
CFSP	4.2 (0.9)	4.1 (0.9)	3.9 (0.8)	3.9 (0.9)	4.1 (0.8)
OFSP	4.1 (0.9)	4.0 (1.0)	3.8 (0.9)	3.9 (0.9)	4.1 (0.9)
*p*-value ^d^	ns	ns	ns	ns	ns

^a^ Mean; ^b^ Standard deviation; ^c^ Friedman’s test; ^d^ The Wilcoxon test; PVA= Provitamin A; CFSP = Cream-fleshed sweet potato; OFSP = Orange-fleshed sweet potato; ns = not significant.

**Table 7:** Significant differences between composite dishes and the sensory attributes of taste, texture and aroma.

Composite A is Preferred over Composite Dish B	Composite Dish A	Composite Dish B	*p*-Value ^a^
Taste	White *phutu* and chicken curry	White *phutu* and bambara groundnut curry	*p* < 0.05
		PVA *phutu* and bambara groundnut curry	*p* < 0.05
	PVA *phutu* and chicken curry	White *phutu* and bambara groundnut curry	*p* < 0.05
		PVA *phutu* and bambara groundnut curry	*p* < 0.05
	White *phutu* and cabbage curry	White *phutu* and bambara groundnut curry	*p* < 0.05
		PVA *phutu* and bambara groundnut curry	*p* < 0.05
	PVA *phutu* and cabbage curry	White *phutu* and bambara groundnut curry	*p* < 0.05
		PVA *phutu* and bambara groundnut curry	*p* < 0.05
	White *phutu* and bambara groundnut curry	PVA *phutu* and bambara groundnut curry	*p* < 0.05
Texture	White *phutu* and chicken curry	White *phutu* and bambara groundnut curry	*p* < 0.05
		PVA *phutu* and bambara groundnut curry	*p* < 0.05
	PVA *phutu* and chicken curry	White *phutu* and bambara groundnut curry	*p* < 0.05
		PVA *phutu* and bambara groundnut curry	*p* < 0.05
	White *phutu* and cabbage curry	White *phutu* and bambara groundnut curry	*p* < 0.05
		PVA *phutu* and bambara groundnut curry	*p* < 0.05
	PVA *phutu* and cabbage curry	White *phutu* and bambara groundnut curry	*p* < 0.05
		PVA *phutu* and bambara groundnut curry	*p*<0.05
Aroma	White *phutu* and cabbage curry	White *phutu* and cabbage curry	*p* < 0.05
		PVA *phutu* and cabbage curry	*p* < 0.05
		White *phutu* and bambara groundnut curry	*p* < 0.05
		PVA *phutu* and bambara groundnut curry	*p* < 0.05
	PVA *phutu* and bambara groundnut curry	White *phutu* and bambara groundnut curry	*p* < 0.05
		PVA *phutu* and bambara groundnut curry	*p* < 0.05
	White *phutu* and cabbage curry	White *phutu* and bambara groundnut curry	*p* < 0.05
		PVA *phutu* and bambara groundnut curry	*p* < 0.05
	PVA *phutu* and cabbage curry	White *phutu* and bambara groundnut curry	*p* < 0.05
		PVA *phutu* and bambara groundnut curry	*p* < 0.05

Composite dish A is preferred over composite dish B for the respective sensory attribute; ^a^ Wilcoxon Test.

**Table 8:** Specific significant differences between composite dishes and the sensory attributes of colour and overall acceptability.

Composite Dish A is Preferred over Composite Dish B	Composite Dish A	Composite Dish B	*p*-Value ^a^
Colour	White *phutu* and chicken curry	White *phutu* and bambara groundnut curry	*p* < 0.05
		PVA *phutu* and bambara groundnut curry	*p* < 0.05
	PVA *phutu* and chicken curry	White *phutu* and cabbage	*p* < 0.05
		White *phutu* and bambara groundnut curry	*p* < 0.05
		PVA *phutu* and bambara groundnut curry	*p* < 0.05
	PVA *phutu* and cabbage curry	White *phutu* and bambara groundnut curry	*p* < 0.05
		PVA *phutu* and bambara groundnut curry	*p* < 0.05
OA	White *phutu* and chicken curry	White *phutu* and bambara groundnut curry	*p* < 0.05
		PVA *phutu* and bambara groundnut curry	*p* < 0.05
	PVA *phutu* and chicken curry	White *phutu* and cabbage curry	*p* < 0.05
		White *phutu* and bambara groundnut curry	*p* < 0.05
		PVA *phutu* and bambara groundnut curry	*p* < 0.05
	White *phutu* and cabbage curry	White *phutu* and bambara groundnut curry	*p* < 0.05
		PVA *phutu* and bambara groundnut curry	*p* < 0.05
	PVA *phutu* and cabbage curry	White *phutu* and bambara groundnut curry	*p* < 0.05
		PVA *phutu* and bambara groundnut curry	*p* < 0.05

Composite dish A is on average significantly better than composite dish B for the respective sensory attribute; ^a^ Wilcoxon Test; OA: Overall acceptability.

**Table 9:** Significant differences in the acceptability ratings of composite dishes across age groups.

Composite Dishes	Sensory Attribute	*p*-Value ^a^	Specific Difference across Age ^b^
White *phutu* and cabbage curry	Taste	<0.05 ^c^	60+ (A) rated higher than 50–59 (B)
White *phutu* and bambara groundnut curry	Taste	<0.05 ^c^	60+ (A) rated higher than 20–29 (B)
White *phutu* and bambara groundnut curry	Aroma	<0.05 ^d^	60+ (A) rated higher than 50–59 (B)
White *phutu* and bambara groundnut curry	OA	<0.05 ^d^	60+ (A) rated higher than 20–29 (B)

^a^ Result from testing for differences across all age groups regarding the specific product and sensory attribute; ^b^ Indicates the age category (years) (A) that gave a statistically higher rating for the respective composite dish and sensory attribute than age category (years) (B); ^c^ ANOVA test; ^d^ Welch test; OA: Overall acceptability.

**Table 10:** Variation in paired preference with gender (*n* = 120).

Gender	*Phutu* and Chicken Curry		*Phutu* and Cabbage Curry		*Phutu* and Bambara Groundnut Curry		Sweet Potato	
	White *Phutu* and Chicken Curry	PVA *Phutu* and Chicken Curry	White *Phutu* and Cabbage Curry	PVA *Phutu* and Cabbage Curry	White *Phutu* and Bambara Groundnut Curry	PVA *Phutu* and Bambara Groundnut Curry	CFSP	OFSP
Males	19 ^a^ (56) ^b^	15 (44)	17 (50)	17 (50)	19 (56)	15 (44)	14 (41)	20 (59)
Females	40 (46)	46 (54)	44 (51)	42 (49)	47 (55)	39 (45)	41 (48)	45 (52)
Total no. participants	59 (49) ^c^	61 (51)	61 (51)	59 (49)	66 (55)	54 (45)	55 (46)	65 (54)

^a^ Number of participants; ^b^ Percentage (%) of the sample within a gender group; ^c^ Percentage (%) of the total number of participants; PVA = Provitamin A; CFSP = Cream-fleshed sweet potato; OFSP = Orange-fleshed sweet potato.

**Table 11:** Preference across age groups (*n* = 120).

Age Group (Years)	*Phutu* and Chicken Curry		*Phutu* and Cabbage Curry		*Phutu* and Bambara Groundnut Curry		Sweet Potato	
	White *Phutu* and Chicken Curry	PVA *Phutu* and chicken curry	White *Phutu* and Cabbage Curry	PVA *phutu* and Cabbage Curry	White *Phutu* and Bambara Groundnut Curry	PVA *Phutu* and Bambara Groundnut Curry	CFSP	OFSP
20–29	7 ^a^ (50) ^b^	7 (50)	9 (64)	5 (36)	9 (64)	5 (36)	5 (36)	9 (64)
30–39	7 (29)	17 (71) ^c^	11 (46)	13 (54)	13 (54)	11 (46)	10 (42)	14 (58)
40–49	11 (39)	17 (61)	12 (43)	16 (57)	16 (57)	12 (43)	14 (50)	14 (50)
50–59	17 (68)	8 (32)	11 (44)	14 (56)	13 (52)	12 (48)	10 (40)	15 (60)
60+	17 (59)	12 (41)	18 (62)	11 (38)	15 (52)	14 (48)	16 (55)	13 (45)
Total no. participants	59 (49)	61 (51)	61 (51)	59 (49)	66 (55)	54 (45)	55 (46)	65 (54)

^a^ Number of participants; ^b^ Percentage (%); ^c^ Bold values within the same column are significantly different at *p* < 0.05 (Pearson Chi-square); PVA = Provitamin A; CFSP = Cream-fleshed sweet potato; OFSP = Orange-fleshed sweet potato.

**Table 12:** Participants’ perceptions towards the consumption of OFSP and PVA-biofortified *phutu* with chicken curry, cabbage curry and bambara groundnut curry.

Themes	Concepts	Quotes	Discussion
Consumer perceptions about yellow maize food combinations and OFSP	Preference of combinations: *Phutu* and chicken Phutu and cabbage Phutu and Bambara groundnut OFSP	‘Yellow *phutu* and chicken was nice.’ ‘Cabbage and yellow *phutu* went good together.’ ‘I did not like this beans.’ ‘This type of beans was different from what I am used to. I love it.’ ‘Orange sweet potato taste nice.’ ‘These beans must be mixed with dry mealies to make iznkobe. It will taste better.’	The FGDs indicated that participants had positive perceptions of the PVA *phutu* when served with chicken curry or cabbage curry. However, they had mixed perceptions when served with bambara groundnut curry. The older FGDs participants perceived that some of the combinations such as phutu and bambara groundnut would not be acceptable to younger consumers, as they were not accustomed to bambara groundnut.
	Food preparation methods	‘Yellow maize could have been cooked for longer.’ ‘Too much water in the orange sweet potato.’ ‘Beans should be cooked with the maize for more flavour.’ ‘Beans could be cooked for longer.’ ‘Chicken would of tasted better with stiff pap.’ ‘Food cooked like I cook at home.’	Participants suggested names of other dishes that could be better accepted. Stiff pap was one of the suggestions given by FGD participants. Although there were mixed responses concerning bambara groundnut, participants offered a few suggestions to improve the acceptability. Participants would have preferred the bambara groundnut to be cooked for a longer period or cooked together with the maize meal.
Cultural acceptance of yellow maize food combinations and OFSP	Expectations of sensory qualities: Smell Appearance Taste Texture	‘Foods were made like I eat at home.’ ‘Our kids may not accept the preparation of the food as it has less oil and spice.’ ‘Thought the orange sweet potato was butternut.’	The majority of the FGDs participants perceived the foods as culturally acceptable; however, they felt that some foods would not be as acceptable to their children and grandchildren. Foods that the younger generation like are prepared with more oil, salt and spices. Most of the FGDs participants perceived the OFSP as butternut due to its orange colour, sweet taste and visual appeal and enjoyed the taste.
Comparison with white maize food combinations and creamed fleshed sweet potato	Expectations of sensory qualities: Smell Appearance Taste Texture	‘Preferred the white sweet potato as too much water in the orange one.’ ‘The orange sweet potato was more nice as it had an orange colour and taste sweet.’ ‘The chicken and yellow maize and beans and yellow maize looked nice.’ ‘First time I had this yellow *phutu* and it was very nice with the cabbage and meat. I won’t eat it alone.’ ‘I did not like the smell of yellow maize.’	Participants would have preferred if OFSP contained less water in comparison to CFSP. However, they found the sweet taste and orange colour of the OFSP very appealing and preferred it to the CFSP. The smell of PVA-biofortified *phutu* and bambara groundnut curry and PVA-biofortified *phutu* on its own were disliked by some of the participants. On the contrary, PVA-biofortified maize was well accepted with the cabbage curry and chicken curry.
Willingness to use yellow maize and OFSP for human consumption	Affordability Availability **Accessibility**	‘Not accessible, if it was I would buy yellow maize and OFSP.’ ‘We use yellow maize in drought times.’ ‘It is fed to animals.’ ‘People are not familiar with yellow maize but will buy if educated on it.’ ‘I would plant if I get seeds.’	Although some participants reported that yellow maize was used to feed animals and eaten during times of drought, the participants expressed a willingness to grow and purchase the PVA-biofortified maize and PVA-biofortified OFSP if planting materials were made available or if the two types of biofortified crops were available as food in the market. The acceptance of PVA-biofortified maize could be improved by educating people on the nutritional properties of PVA-biofortified crops and preparation methods used to cook these crops.

## Supplementary Materials

The following are available online at https://www.mdpi.com/2072-6643/11/7/1577/s1.
